# A novel interaction between CX_3_CR_1_ and CCR_2_ signalling in monocytes constitutes an underlying mechanism for persistent vincristine-induced pain

**DOI:** 10.1186/s12974-018-1116-6

**Published:** 2018-04-06

**Authors:** Karli Montague, Raffaele Simeoli, Joao Valente, Marzia Malcangio

**Affiliations:** 1 Wolfson Centre for Age-Related Diseases, King’s College London, Guy’s Campus, London, SE1 1UL UK; 2 Infectology and Clinical Trials Research Department, Bambino Gesu` Children’s Hospital, IRCCS, Rome, Italy; 3 Vascular Biology and Inflammation Section, Cardiovascular School of Medicine & Science, British Heart Foundation Centre of Excellence, King’s College London, Franklin-Wilkins Building, 150 Stamford Street, King’s College London, London, SE1 9NH UK

**Keywords:** Vincristine, CIPN, CX_3_CR_1_, CCL_2_, CCR_2_, Monocyte, Macrophage, Sciatic nerve, THP-1, Proinflammatory cytokine

## Abstract

**Background:**

A dose-limiting side effect of chemotherapeutic agents such as vincristine (VCR) is neuropathic pain, which is poorly managed at present. Chemokine-mediated immune cell/neuron communication in preclinical VCR-induced pain forms an intriguing basis for the development of analgesics. In a murine VCR model, CX_3_CR_1_ receptor-mediated signalling in monocytes/macrophages in the sciatic nerve orchestrates the development of mechanical hypersensitivity (allodynia). CX_3_CR_1_-deficient mice however still develop allodynia, albeit delayed; thus, additional underlying mechanisms emerge as VCR accumulates. Whilst both patrolling and inflammatory monocytes express CX_3_CR_1_, only inflammatory monocytes express CCR_2_ receptors. We therefore assessed the role of CCR_2_ in monocytes in later stages of VCR-induced allodynia.

**Methods:**

Mechanically evoked hypersensitivity was assessed in VCR-treated CCR_2_- or CX_3_CR_1_-deficient mice. In CX_3_CR_1_-deficient mice, the CCR_2_ antagonist, RS-102895, was also administered. Immunohistochemistry and Western blot analysis were employed to determine monocyte/macrophage infiltration into the sciatic nerve as well as neuronal activation in lumbar DRG, whilst flow cytometry was used to characterise monocytes in CX_3_CR_1_-deficient mice. In addition, THP-1 cells were used to assess CX_3_CR_1_-CCR_2_ receptor interactions in vitro, with Western blot analysis and ELISA being used to assess expression of CCR_2_ and proinflammatory cytokines.

**Results:**

We show that CCR_2_ signalling plays a mechanistic role in allodynia that develops in CX_3_CR_1_-deficient mice with increasing VCR exposure. Indeed, the CCR_2_ antagonist, RS-102895, proves ineffective in mice possessing functional CX_3_CR_1_ receptors but reduces VCR-induced allodynia in CX_3_CR_1_-deficient mice, in which CCR_2_^+^ monocytes are elevated by VCR. We suggest that a novel interaction between CX_3_CR_1_ and CCR_2_ receptors in monocytes accounts for the therapeutic effect of RS-102895 in CX_3_CR_1_-deficient mice. Indeed, we observe that CCR_2_, along with its ligand, CCL_2_, is elevated in the sciatic nerve in CX_3_CR_1_-deficient mice, whilst in THP-1 cells (human monocytes), downregulating CX_3_CR_1_ upregulates CCR_2_ expression via p38 MAP kinase signalling. We also show that the CX_3_CR_1_-CCR_2_ interaction in vitro regulates the release of pronociceptive cytokines TNF-α and IL1β.

**Conclusions:**

Our data suggests that CCL_2_/CCR_2_ signalling plays a crucial role in VCR-induced allodynia in CX_3_CR_1_-deficient mice, which arises as a result of an interaction between CX_3_CR_1_ and CCR_2_ in monocytes.

**Electronic supplementary material:**

The online version of this article (10.1186/s12974-018-1116-6) contains supplementary material, which is available to authorized users.

## Background

Chemotherapy-induced painful neuropathy (CIPN) is a dose-limiting side effect of chemotherapeutic drugs, which is poorly managed by analgesics at present [1]. Current treatments for CIPN such as gabapentin and opioids show limited efficacy and engender a host of undesirable side effects such as dizziness and nausea [2, 3]. Indeed, it has been reported that in as many as 40% of patients [4], CIPN results in the premature cessation of chemotherapy and thus jeopardises cancer treatment success. It is therefore essential that novel, more efficacious analgesics are developed and tailored according to different chemotherapeutic agents, which have a broad range of actions. This warrants a deeper understanding of the underlying drug-specific mechanisms of CIPN.

Most chemotherapeutic agents do not cross the blood-brain barrier but are able to penetrate the blood-nerve barrier, where they accumulate in dorsal root ganglia (DRG) and peripheral nerves, causing toxicity that is intensified by the absence of a lymphatic system in the endoneurial compartment [5–7]. Treatments that are currently used for CIPN are predominantly ‘neurocentric’ therapies, which target neuronal responses to injury inflicted by chemotherapeutic agents. Compelling evidence from preclinical models of CIPN in recent years however has uncovered the importance of immune cells, such as monocytes/macrophages, and their communication at the endothelial-neuronal interface peripherally [8, 9].

One chemotherapeutic agent associated with neuropathic pain is the vinca alkaloid, vincristine (VCR), used most commonly in the treatment of lymphomas and leukaemias [10]. In a preclinical model of VCR-induced pain, VCR-mediated activation of the endothelium promotes the infiltration of CX_3_CR_1_-expressing monocytes into peripheral nerves, where they differentiate into macrophages and activate TRPA_1_ channels on sensory neurons via the release of reactive oxygen species [9]. An increase in monocyte infiltration into the sciatic nerve is causative of mechanical hypersensitivity (allodynia), as opposed to being an epiphenomenon or downstream event, on the basis that transient depletion of macrophages delays the onset of allodynia until such depletion is ceased and macrophages can populate the sciatic nerve [9]. Communication between monocytes/macrophages and sensory neurons in the periphery has been found to be a key underlying mechanism in preclinical CIPN associated with other chemotherapeutic agents such as paclitaxel. Indeed, depletion of macrophages in the DRG prevents paclitaxel-induced allodynia [11, 12]. Furthermore, inhibition of CX_3_CR_1_-mediated activation of macrophages specifically, as well as inhibition of p38 MAPK, a downstream target of CX_3_CR_1_ activation, also blocks the development of paclitaxel-induced allodynia [11].

Despite the promise of targeting CX_3_CR_1_ receptors in monocytes/macrophages as a prophylaxis for VCR pain, and, indeed, pain associated with other chemotherapeutic agents, a critical caveat remains: CX_3_CR_1_-deficient mice still develop VCR-induced allodynia, albeit delayed [9]. It therefore appears that the role of CX_3_CR_1_ in monocytes is dynamic and alternative mechanisms emerge with increased exposure to VCR. In this study, we attempt to elucidate such a mechanism that could account for the eventual onset of VCR-induced allodynia in CX_3_CR_1_-deficient mice.

Circulating monocytes can, very broadly speaking, exist as two phenotypes—‘patrolling’ or ‘inflammatory’, each of which expresses distinct markers [13]. Patrolling monocytes, characterised by low expression of Ly6C (Ly6C^low^) and high levels of CX_3_CR_1_ (CX_3_CR_1_^high^), are predominantly associated with homeostatic functions [14]. Inflammatory or ‘classical’ monocytes however express high levels of Ly6C (Ly6C^high^) and relatively low levels of CX_3_CR_1_ (CX_3_CR_1_^low^). Unlike patrolling monocytes, inflammatory monocytes express the chemokine receptor CCR_2_ (CCR_2_^+^). In the sciatic nerve, CCR_2_ receptor is activated by CCL_2_, which is expressed by endothelial cells, monocytes/macrophages and sensory neurons [15–17]. Inflammatory monocytes are recruited to sites of injury and inflammation in a CCL_2_/CCR_2_-regulated fashion, releasing a multitude of factors, many of which exert pronociceptive functions [18]. It is well-established that neuronal injury, such as that caused by chemotherapy, is accompanied by an increase in macrophages with the inflammatory CCR_2_^+^ phenotype [19, 20].

In this study, we therefore investigate the role of CCL_2_/CCR_2_ signalling in inflammatory monocytes/macrophages in VCR-induced allodynia—specifically at later stages of treatment, when the role of CX_3_CR_1_ is less pertinent. Given that the CCR_2_ receptor is also expressed in sensory neurons [17], we also consider the role of CCR_2_-mediated neuronal activation in order to differentiate between the roles of CCR_2_ signalling in sensory neurons and monocytes. As well as attempting to elucidate the role of CCR_2_^+^ monocytes/macrophages in VCR-induced allodynia in CX_3_CR_1_-deficient mice, we also investigate a potential interaction between CX_3_CR_1_ and CCR_2_ expression in immortalised human monocytes (THP-1 cells). Furthermore, we assess the regulation of pronociceptive cytokines from THP-1 cell downstream of the CX_3_CR_1_-CCR_2_ interaction, which could constitute a potential monocyte-derived signal that mediates monocyte communication with nociceptive neurons, which are known to express cytokine receptors [21].

## Methods

### Animals

Experiments were performed in accordance with the United Kingdom Animals (Scientific Procedures) Act 1986 and local animal care and use guidelines. Male and female mice (25–30 g, 12–17 weeks of age) were used. They did not differ with respect to thresholds. Mice were randomly assigned to groups. Each group contained approximately equal numbers of age-matched mice of both sexes.

For Cx3cr1-gfp (green fluorescent protein) mice, an original breeding stock was a kind gift from Steffen Jung (Weizmann Institute of Science, Israel). Cx3cr1-gfp heterozygous mice were generated and genotyped as described [9]. For Ccr2-rfp (red fluorescent protein) mice, an original breeding stock was purchased from Jackson Laboratory. Ccr2 disruption/RFP expression was confirmed by PCR using published primers [22]. All mice used were generated from a C57Bl/6 background.

### Behavioural testing

Static mechanical withdrawal thresholds were assessed as described previously [9]. Briefly, unrestrained animals were acclimatised for 45 min prior to testing. Calibrated von Frey filaments (0.008–1.4 g) were applied to the plantar surface of the hind paw until they bent and were held for 3 s or until paw withdrawal. A 50% paw withdrawal threshold (in grams) was calculated using the ‘up-down’ method starting with the 0.6-g filament. Three baseline (B) measurements were made prior to treatment, the mean of which is presented. Bilateral measurements were made with no difference in threshold being recorded between left and right (data not shown). Data presented are means of the left and right hind paw thresholds. All tests were conducted blind.

### Drug administration

Vincristine sulphate (VCR; Sigma-Aldrich) was dissolved in sterile saline. 0.5 mg/kg/day VCR was injected intraperitoneally (i.p.) using a 25-g needle for two 5-day cycles (days 0–4 and 7–11) with a 2-day break as previously described [9]). RS-102895 hydrochloride (Sigma), which is small molecule antagonist of CCR_2_ receptor belonging to the spiro-piperidine class, was dissolved in dimethyl sulphoxide (DMSO) at 10 mg/ml and diluted in sterile saline; 20 mg/kg/day was injected i.p. for 5 days [23] during the second VCR cycle.

### Immunohistochemistry

Mice, under pentobarbital anaesthesia, were transcardially perfused with saline, followed by 4% paraformaldehyde (PFA). The lumbar enlargement, bilateral L3–5 DRG, and sciatic nerves were excised. Tissue was post-fixed for 4 h and then dehydrated in 30% sucrose in PBS before being embedded in optimum cutting temperature-embedding medium (VWR) and frozen on dry ice. Fifteen-micrometer cryosections were mounted onto Superfrost Plus slides (VWR). Sections were permeabilised for 15 min at room temperature in 0.1% PBS-Triton X-100 (Sigma-Aldrich). Slides were blocked in 0.1% PBS-Triton X-100 + 3% BSA (Sigma-Aldrich) for 1 h at room temperature before being incubated in primary antibody at 4 °C for 16 h. Slides were washed thrice in 0.1% PBS-Triton X-100, before being incubated with the appropriate fluorescently tagged secondary antibody for 1.5 h at room temperature. Slides were washed again and then mounted using FluorSave™ (Merck, UK) and visualised under a Zeiss LSM710 confocal microscope (Zeiss). Five sections per mouse were randomly selected and analysed blind as described previously [9].

### Antibodies

To visualise microglia, anti-Iba1 (1:100, WAKO) and anti-rabbit Alexa Fluor 488 (1:1000, Invitrogen) were used. For F4/80 expression, anti-F4/80 (1:400, Abcam) was used followed by anti-rat Alexa 488 or 568 (both 1:1000, Invitrogen). For CCL_2_ in the sciatic nerve, anti-CCL_2_ (1:200, Invitrogen) and anti-rabbit Alexa 568 (1:1000, Invitrogen) were used. P-ERK visualisation in DRG was carried out using anti-p-ERK (1:300, Cell Signaling Technology) followed by anti-rabbit Alexa 568 (1 in 1000 Invitrogen). GFP did not require amplification. In all cases, control staining was performed in which no primary antibody was applied.

### Western blot

Sciatic nerves were fresh-dissected, snap-frozen and stored at − 80 °C. Tissue was homogenised in ice-cold RIPA buffer (20 mM tris(hydroxymethyl)aminomethane; 10 mM NaF; 150 mM NaCl; 1% nonyl-phenoxylpolyethoxylethanol; 1 mM phenylmethanesulfonyl fluoride; 1 mM Na_3_VO_4_ (all Sigma-Aldrich); and 10 mg/ml proteinase inhibitor (Roche)) before being incubated at 4 °C with agitation for 2 h. Samples were centrifuged at 4 °C and 13,000 rpm for 20 min. Supernatant was used for Western blot. For THP-1 cultures, medium was spun at 2000*g* for 5 min at 4 °C. The pellet was washed, re-suspended in radioimmunoprecipitation assay (RIPA) buffer, vortexed and kept on ice for 30 min, and then spun at 13,000 rpm at 4 °C for 10 min. The supernatant was collected. Protein concentration was determined using the bicinchoninic acid (BCA) protein assay (Pierce). Twenty micrograms of protein per sample was separated on a 15% SDS-PAGE gel and transferred onto a nitrocellulose membrane which was performed according to the manufacturer’s instructions (Bio-Rad, UK). Blots were blocked for 1 h at room temperature in 0.1% TBS-Tween-20 (TBST) + 5% skimmed milk before being incubated in primary antibody (F4/80), CCL_2_ (anti-mouse or human), CCR_2_ (anti-mouse or human) (all 1:1000, Abcam) and p-ERK (1:1000 Cell Signaling Technology) at 4 °C overnight along with a loading control (α-tubulin, β-actin both 1:1000, Abcam). After washing in 0.1% TBST, blots were incubated for 1 h at room temperature with HRP-conjugated secondary antibody (1:2000, DAKO). Blots were washed before being developed with SuperSignal™ West Femto substrate (Thermo Fisher Scientific). Bands were visualised using BioSpectrum© Imaging and quantified using Quantity One (Bio-Rad, UK).

### Flow cytometry

Mice were sacrificed by a rising concentration of CO_2_. Peritoneal cells were then elicited by injecting saline into the peritoneal cavity. Lavages were collected and centrifuged at 2400 rpm for 10 min. Pellets were suspended in red phenol-free DMEM (Gibco) supplemented with 10% heat-inactivated foetal bovine serum (HI-FBS), 1% pen/strep and 1% sodium pyruvate. Cells were incubated with conjugated antibodies (CD45.1-Pacific Blue (BioLegend); F4/80-PE (eBioscience); Alexa Fluor® 647 anti-mouse CD192 (CCR_2_) Antibody (BioLegend); Ly-6C APC (eBioscience)) on ice for 30 min and then washed twice with PBS containing 1.5% BSA before being re-suspended prior to analysis. Cells were gated and analysed with LSRFortessa™ (BD bioscience) and FlowJo software (Tree Start).

### THP-1 culture

THP-1 cells were a kind gift from Mauro Perretti (Queen Mary, University of London). Cells were cultured in RPMI 1640 with 10% FBS and 2 mM glutamine (Sigma-Aldrich) and maintained in upright T25 tissue culture flasks. For all experiments, cells were cultured in 24-well plates. Cells were transfected using Lipofectamine RNAiMAX (Thermo Fisher Scientific) with CX_3_CR_1_ siRNA (Insight Biotechnology) or control SignalSilence®siRNA (New England Biolabs) 24 h after seeding, and assays were performed 48 h post-transfection. Efficiency of CX_3_CR_1_ knockdown was determined using Western blot. In some experiments, cells were pre-treated, 1 h before transfection with siRNA, with SB203580 or PD98059 (both 25 μM, Cambridge Bioscience). For CCL_2_ stimulation, cells were treated for 3 h with human recombinant CCL_2_ (Cambridge Bioscience) at 10, 50 and 100 ng/ml in HEPES buffer.

### Cytokine/chemokine measurement

CCR_2_ expression was measured using human chemokine C-C-motif receptor 2 (CCR_2_) ELISA Kit (2B Scientific) according to the manufacturer’s instructions. For cytokine expression, a multi-analyte ELISA for proinflammatory cytokines (Qiagen) was performed on culture medium to assess which cytokines were upregulated. ELISA was then performed specifically for TNFα and IL1β (Abcam) according to the manufacturer’s instructions.

### Statistics

All data were analysed using SPSS (IBM Analytics). Behavioural data were analysed by repeated measures (RM) two-way ANOVA, followed by Tukey’s test. All other in vivo data were analysed by one-way ANOVA, followed by Tukey’s test. All in vitro data was analysed using Student’s paired *t* test. All data are shown as mean ± SEM, and data were considered significant when *p* < 0.05.

## Results

### CCR_2_-deficient mice develop reduced allodynia relative to CCR_2_ heterozygous controls at higher cumulative doses of vincristine

**Fig. 1:**
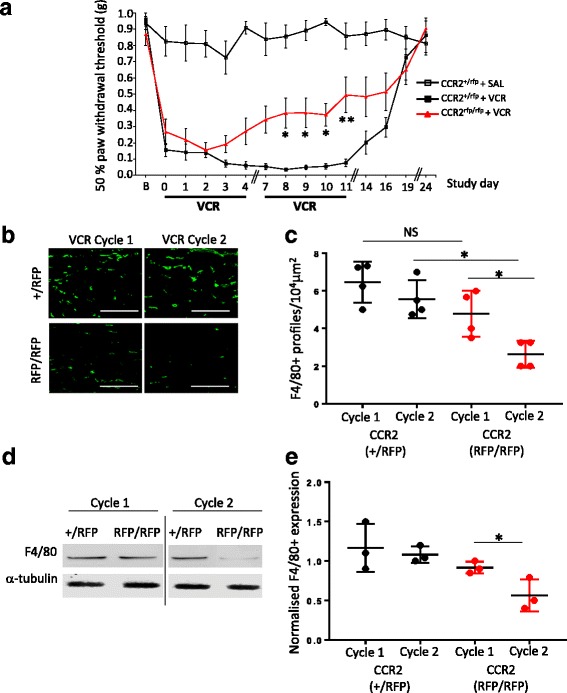
CCR_2_-deficient mice have reduced VCR allodynia and monocyte infiltration in the sciatic nerve during the second cycle. **a** Mechanical thresholds are significantly higher in VCR-treated CCR_2_-deficient (RFP/RFP) mice than heterozygous controls during the second cycle (days 8–11). Data expressed as 50% paw withdrawal thresholds (mean ± SEM, *n* = 8 mice per group). *p < 0.05 and **p < 0.01 compared to time-matched VCR-treated CCR_2_^+/RFP^ thresholds, two-way RM ANOVA, post hoc Tukey’s test. **b** Representative images showing macrophages (F4/80, green) in longitudinal sections of sciatic nerves of CCR_2_^+/RFP^ and CCR_2_^RFP/RFP^ mice during both VCR cycles. Scale bar = 200 μm. **c** Quantification of F4/80 immunoreactive (+) profiles per 10^4^ μm^2^ (mean ± SEM, *n* = 4 mice per group; five fields of view were quantified for each mouse). There is a significant reduction in F4/80+ profiles in CCR_2_^RFP/RFP^ sciatic nerves in cycle 2, relative to CCR_2_^RFP/RFP^ sciatic nerves during cycle 1 and relative to CCR2^+/RFP^ during cycle 2. *p < 0.05, one-way ANOVA, Tukey’s test. **d** Representative blot of F4/80 (130 kDa) and α-tubulin loading control (50 kDa) in sciatic nerve homogenates obtained from CCR_2_^+/RFP^ and CCR2^RFP/RFP^ mice during VCR cycles 1 and 2. **e** Quantification of F4/80 band density normalised to α-tubulin (mean ± SEM, *n* = 3). F4/80 expression is unchanged in CCR_2_^+/RFP^ sciatic nerve homogenates between VCR cycles 1 and 2. In CCR_2_^RFP/RFP^ sciatic nerve homogenates, there is a significant reduction in normalised F4/80 expression in cycle 2 (day 11) relative to cycle 1 (day 4). *p < 0.05, one-way ANOVA, Tukey’s test

In order to establish whether CCR_2_ receptor signalling plays a role in VCR-induced allodynia, CCR_2_-deficient mice (CCR_2_^RFP/RFP^) were treated with two 5-day cycles of VCR [9, 24]. In the first cycle, they were found to develop significant VCR-induced allodynia to the same severity and within the same time frame as heterozygous (CCR_2_^+/RFP^), littermate controls (Fig. 1a). Specifically, within 24 h of the first VCR dose and throughout the first treatment cycle, mechanical withdrawal thresholds of both CCR_2_-deficient and heterozygous mice were significantly reduced relative to saline-treated mice. Indeed, there was no difference in threshold between VCR-treated CCR_2_-deficient and heterozygous mice throughout the first VCR cycle (days 0–4). During the second cycle of VCR however, CCR_2_-deficient mice had significantly higher thresholds, thus reduced allodynia, relative to heterozygous controls (Fig. 1a).

### VCR-induced monocyte infiltration in peripheral nerve tissue is significantly reduced in CCR_2_-deficient mice during the second VCR cycle

We initially confirmed that VCR administration did not cause a measurable microglial response (Additional file 1: Figure S1A, B), before focussing our attention on the sciatic nerve, where VCR accumulates and mechanisms underlying preclinical VCR pain are most pertinent [9]. In CCR_2_ heterozygous mice, the elevation of macrophages in the sciatic nerve occurred to the same degree during both the first and second cycles of VCR treatment as demonstrated by immunohistochemical (Fig. 1b, c) and Western blot detection (Fig. 1d, e) of the macrophage marker F4/80. In CCR_2_-deficient mice however, when allodynia was significantly reduced during the second cycle relative to the first cycle, we observed that F4/80 expression was also significantly reduced in the sciatic nerve relative to the first VCR cycle (Fig. 1b–e).

### Treatment of CX_3_CR_1_ heterozygous mice with a CCR_2_ antagonist does not reduce VCR-induced allodynia or monocyte infiltration in the sciatic nerve

In light of the apparent involvement of the CCR_2_ receptor in VCR allodynia during the second VCR cycle, we wanted to establish if treatment with a CCR_2_ antagonist on VCR-induced allodynia mirrors genetic deficiency, as well as identify whether CCR_2_ does indeed play a role in VCR-induced allodynia in CX_3_CR_1_-deficient mice during the second cycle. To address the first of these questions, we began by administering the CCR_2_ antagonist RS-102895 during the second VCR cycle to CX_3_CR_1_ heterozygous mice, which possess functional CX_3_CR_1_. Our rationale for therapeutic (VCR cycle 2) as opposed to prophylactic (VCR cycle 1) dosing was derived not only from the apparent effect of CCR_2_ deficiency on VCR-induced allodynia during the second VCR cycle specifically but also from previous observations that whilst the development of VCR-induced allodynia is orchestrated by CX_3_CR_1_ signalling in monocytes, other mechanisms appear to regulate allodynia during the second VCR cycle [9]. Indeed, prophylactic treatment with RS-102895 during the first VCR cycle had no effect on withdrawal thresholds (Additional file 1: Figure S2). However, we found that when CX_3_CR_1_ heterozygous mice were given RS-102895 during the second VCR cycle, there was still no change in VCR-induced allodynia (Additional file 1: Figure S3A). Concurrently, VCR-induced monocyte/macrophage infiltration into the sciatic nerve was also unaltered by administration of RS-102895 during the second VCR cycle (Additional file 1: Figure S3B, C).

### Treatment of CX_3_CR_1_-deficient mice with a CCR_2_ antagonist significantly reduces VCR-induced allodynia and monocyte infiltration into peripheral nervous tissue

**Fig. 2:**
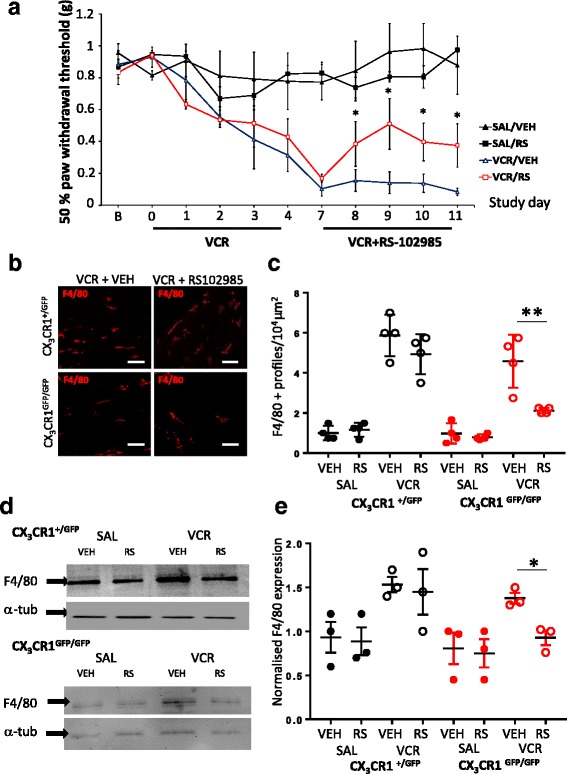
RS-102895 significantly reduces VCR-induced allodynia and monocyte infiltration in the sciatic nerve in CX_3_CR_1_-deficient mice. **a** CX_3_CR_1_ knockout (GFP/GFP) mice were treated with two VCR cycles alongside the CCR_2_ antagonist RS-102985, or vehicle, during the VCR cycle 2. CX_3_CR_1_^GFP/GFP^ mice developed VCR-induced allodynia at the end of cycle 1 (day 4) as previously reported. Data expressed as 50% paw withdrawal thresholds (mean ± SEM, *n* = 6–9 mice per group). In VCR/RS-102895-treated CX_3_CR_1_^GFP/GFP^ mice (red trace), withdrawal thresholds increased significantly relative to VCR/vehicle-treated CX_3_CR_1_^GFP/GFP^ mice (blue) within 24 h of the first antagonist dose. *p < 0.05, two-way RM ANOVA, Tukey’s test. **b** Representative images of macrophages (F4/80, red) in sciatic nerve longitudinal sections at day 11 from VCR-treated CX_3_CR_1_^GFP/GFP^ and CX_3_CR_1_^+/GFP^ mice. Scale bar = 50 μm. **c** Quantification of F4/80+ profiles per 10^4^ μm^2^ in the sciatic nerve at day 11 (mean ± SEM, *n* = 4 mice per group; five fields of view were quantified for each mouse). RS-102985 significantly reduces VCR-induced elevation of F4/80+ profiles in sciatic nerves from CX_3_CR_1_^GFP/GFP^ mice. NS not significant, **p < 0.01, one-way ANOVA, Tukey’s test. **d** Representative blot of F4/80 (130 kDa) and α-tubulin (50 kDa) in sciatic nerve homogenates obtained from CX_3_CR_1_^+/GFP^ and CX_3_CR_1_^GFP/GFP^ mice at day 11. **e** Quantification of F4/80 band density normalised to α-tubulin (mean ± SEM, *n* = 3). RS-102985 significantly reduces VCR-associated infiltration into sciatic nerves obtained from CX_3_CR_1_^GFP/GFP^ mice. NS not significant, *p < 0.05, one-way ANOVA, post hoc Tukey’s test

Although our data so far suggests that CCR_2_ antagonism during the second VCR cycle is not necessarily therapeutic in mice possessing functional CX_3_CR_1_ receptor, this does not rule out a role for CCR_2_-mediated signalling in VCR-induced allodynia that has been observed in CX_3_CR_1_-deficient mice at later stages of treatment [9]. We therefore treated CX_3_CR_1_-deficient (CX_3_CR_1_^GFP/GFP^) mice with two cycles of VCR in the presence of RS-102895 (or vehicle) during the second VCR cycle, when allodynia manifests in CX_3_CR_1_-deficient mice [9]. As observed previously, CX_3_CR_1_-deficient mice displayed a delayed onset of allodynia in response to VCR treatment relative to heterozygous littermates. Whilst CX_3_CR_1_ heterozygous mice developed significant allodynia within 24 h of the first VCR dose (Additional file 1: Figure S3A), mechanical thresholds only dropped significantly in CX_3_CR_1_-deficient mice relative to saline-treated controls at the end of the first VCR cycle (Fig. 2a). In addition, dissimilar to the results obtained in CX_3_CR_1_ heterozygous mice, systemic treatment of CX_3_CR_1_-deficient mice with RS-102895 during the second VCR cycle significantly reduced VCR-induced allodynia (Fig. 2a). Although withdrawal thresholds in CX_3_CR_1_-deficient mice treated with RS-102895 alongside VCR were still significantly lower than baseline levels, a significant increase relative to CX_3_CR_1_-deficient mice treated with VCR alone was observed within 24 h of the first RS-102895 dose and persisted throughout the second VCR cycle. Crucially, administration of RS-102895 in all saline-treated mice did not alter mechanical thresholds (Fig. 2a, Additional file 1: Figure S3). This data exposes a potential role for CCL_2_/R_2_ signalling in VCR-induced allodynia in CX_3_CR_1_-deficient mice during the second cycle specifically.

Given that RS-102895 significantly reduced VCR-induced allodynia in CX_3_CR_1_-deficient mice during the second VCR cycle, we assessed whether it also resulted in a simultaneous reduction in VCR-induced monocyte infiltration into the sciatic nerve. In CX_3_CR_1_ heterozygous mice, RS-102985 administration during the second VCR cycle did not significantly reduce VCR-induced monocyte infiltration into the sciatic nerve, as demonstrated by immunohistochemistry (Fig. 2b, c) and Western blot analysis (Fig. 2d, e) of F4/80. However, the administration of RS-102895 to VCR-treated CX_3_CR_1_-deficient mice did indeed result in a significant reduction in macrophage number in the sciatic nerve (Fig. 2b–e). This suggests that the mechanism by which RS-102895 significantly reduces VCR-induced allodynia in CX_3_CR_1_-deficient mice is, at least in part, regulated by CCR_2_ signalling in, or CCR2-mediated recruitment of, monocytes/macrophages in peripheral nerves. Furthermore, the reduction in VCR-induced allodynia and monocyte infiltration that accompanied the administration of RS-102895 to CX_3_CR_1_-deficient mice specifically is suggestive of changes in CCR_2_ expression and/or activity as a result of loss of CX_3_CR_1_ in monocytes/macrophages in the periphery.

### The reduction in VCR-induced allodynia in CX_3_CR_1_-deficient mice treated with RS-102895 does not require sensory neuron CCR_2_ receptors

**Fig. 3:**
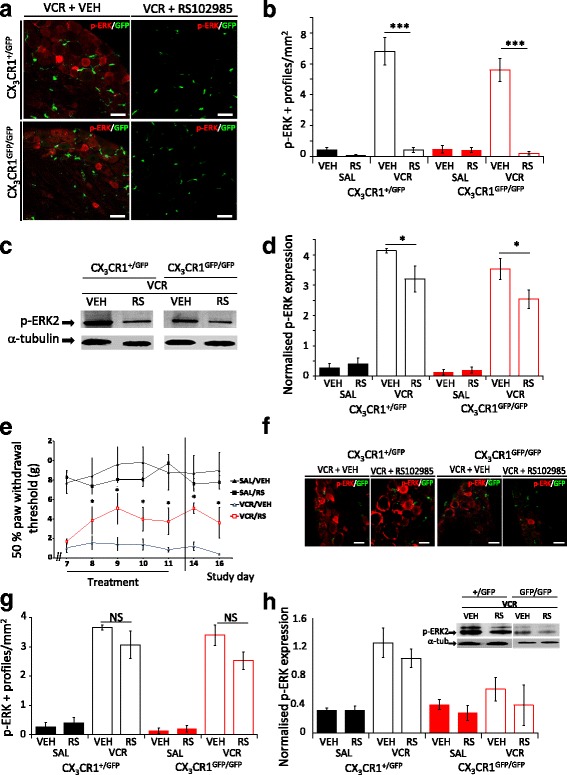
Reduction in VCR-induced allodynia by RS-102985 does not require neuronal activation. **a** Representative images of GFP (macrophages, CX_3_CR_1_, green) and p-ERK (neuronal activation, red) in L4 DRG taken from VCR-treated CX_3_CR_1_^+/GFP^ and CX_3_CR_1_^GFP/GFP^ mice at treatment day 11. Mice were co-treated with RS-102985 or vehicle. Scale bar, 50 μm. **b** Quantification of p-ERK+ profiles per mm^2^ at day 11 (mean ± SEM, *n* = 4 mice per group). VCR induces neuronal activation, which is significantly reduced by RS-102895 at day 11. ***p < 0.001, one-way ANOVA, Tukey’s test. **c** Representative blot of p-ERK2 (42 kDa) and α-tubulin (50 kDa) in L3–L5 DRG homogenates obtained from CX_3_CR_1_^+/GFP^ and CX_3_CR_1_^GFP/GFP^ mice at treatment day 11. **d** Quantification of p-ERK2 band density normalised to α-tubulin (mean ± SEM, *n* = 3). In both genotypes, RS-102895 administration significantly reduces VCR-induced p-ERK2 expression in L3–L5 DRG. *p < 0.05, one-way ANOVA, Tukey’s test. **e** Withdrawal thresholds at days 14–16 remain significantly higher in VCR/RS-102895-treated CX3CR_1_^GFP/GFP^ mice relative to those treated with VCR and vehicle (mean ± SEM, *n* = 6–9 mice per group). *p < 0.05, two-way RM ANOVA, Tukey’s test. **f** Representative images of GFP (macrophages) and p-ERK (red) in L4 DRG taken from CX_3_CR_1_^+/GFP^ and CX_3_CR_1_^GFP/GFP^ mice at day 14. Prior RS-102895 treatment does not continue to prevent p-ERK activation in either genotype. Scale bar = 50 μm. **g** Quantification of p-ERK+ profiles per mm^2^ at day 14 (mean ± SEM, *n* = 4 mice per group). NS not significant. **h** Representative blot and quantification of p-ERK2 and α-tubulin in bilateral L3–L5 DRG homogenates. In both genotypes, DRG from VCR/RS-102895-treated mice express similar normalised p-ERK2 expression to VCR/vehicle-treated mice (mean ± SEM, *n* = 3)

Unlike CX_3_CR_1_, which is predominantly expressed by monocytes/macrophages, CCR_2_ is additionally expressed on sensory neurons [16, 17]. To investigate the potential contribution of CCR_2_ signalling in sensory neurons to the reduction in VCR-induced allodynia observed in RS-102895-treated CX_3_CR_1_-deficient mice, we considered the effect of RS-102895 on CCR_2_-mediated neuronal activation. In both CX_3_CR_1_-deficient and heterozygous mice, two cycles of VCR administration resulted in the upregulation of phospho-p44/42 MAPK2 (p-ERK2), a marker for neuronal activation and downstream target of CCR_2_ signalling [25, 26] as demonstrated by both immunohistochemistry (Fig. 3a, b) and Western blot analysis in lumbar DRG (Fig. 3c, d). Administration of RS-102895 for the duration of the second VCR cycle however resulted in a significant reduction of p-ERK2 in lumbar DRG from both CX_3_CR_1_-deficient and heterozygous mice (Fig. 3a–d) suggesting that the effect of RS-102895 on VCR-induced elevation of p-ERK2 did not differ between genotypes. Crucially, at day 14 (3 days after treatment cessation), VCR-treated CX_3_CR_1_-deficient mice that had received RS-102895 still had significantly reduced allodynia relative to CX_3_CR_1_ heterozygous mice (Fig. 3e). However, p-ERK2 activation was observed in lumbar DRG at this time point regardless of genotype, as demonstrated by immunohistochemistry (Fig. 3f, g) and Western blot analysis (Fig. 3h). This could suggest that the reduction in VCR-associated allodynia in RS-102895-treated CX_3_CR1-deficient mice is not entirely dependent on the diminution of sensory neuronal activation via CCR_2_ receptor antagonism and could therefore also be regulated by CCR_2_ signalling elsewhere, such as that present in monocytes/macrophages.

This data also indicates that it is less likely that the absence of effect of RS-102895 in CX_3_CR_1_ heterozygous mice was due to lack of bioavailability. Activation of p-ERK is a well-established downstream event of CCR_2_ receptor activation; thus, the fact that VCR-induced p-ERK activation was only reduced when RS-102895 was administered and not at day 14, at which point treatment was terminated (Fig. 3e–g), provides us with a measureable outcome of RS-102895 bioavailability and target engagement.

### CCL_2_/R_2_ expression is altered in macrophages in the sciatic nerve of CX_3_CR_1_-deficient mice

**Fig. 4:**
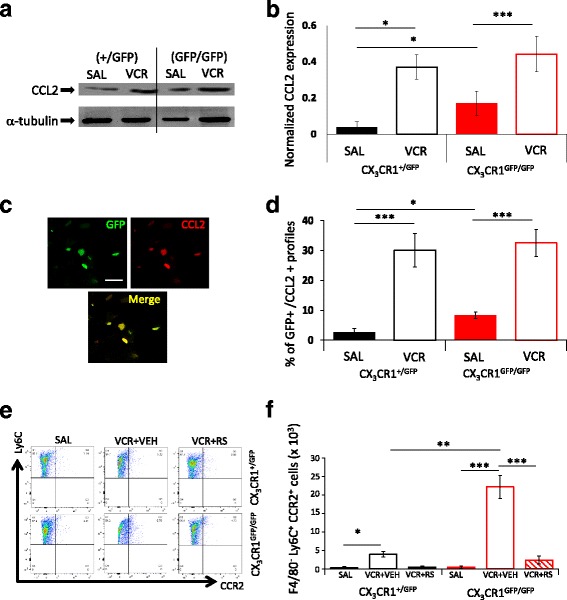
CX_3_CR_1_ deficiency increases CCR_2_ expression in vivo. **a** Representative blot for CCL_2_ (15 kDa) in CX_3_CR_1_^+/GFP^ and CX_3_CR_1_^GFP/GFP^ sciatic nerves. **b** Quantification of CCL_2_ band density normalised to α-tubulin (mean ± SEM, *n* = 3). Basal expression of CCL_2_ in sciatic nerve homogenates is significantly higher in CX_3_CR_1_-deficient mice relative to saline-treated CX_3_CR_1_ heterozygous. VCR significantly increases CCL_2_ in both genotypes. *p < 0.05, ***p < 0.005, one-way ANOVA, Tukey’s test. **c** Representative images of GFP (macrophages) and CCL_2_ (red) in sciatic nerve transverse sections at day 11. Scale bar = 50 μm. **d** Quantification of the percentage of GFP+ profiles that are also CCL_2_+. Two cycles of VCR significantly increases the percentage of GFP+/CCL_2_+ macrophages in both genotypes (mean ± SEM, *n* = 4 mice per group). ***p < 0.005, one-way ANOVA, post hoc Tukey’s test. **e** Representative dot blots for peritoneal lavages. Numbers in the gates refer to percentage of positive cells. **f** Bar chart representing number of F4/80^−^Ly6C^+^CCR2^+^ events (mean ± SEM, *n* = 3). *p < 0.05, **p < 0.01 and ***p < 0.005, one-way ANOVA, Tukey’s test

Given the effect of RS-102895 treatment on CX_3_CR_1_-deficient mice specifically and that CX_3_CR_1_ deficiency is associated with CCL_2_ upregulation in phagocytic cells [27, 28], we went on to examine CCL_2_ expression in the sciatic nerve of CX_3_CR_1_-deficient mice. Western blot analysis of CCL_2_ expression in sciatic nerve homogenates from CX_3_CR_1_-deficient and heterozygous mice that had been treated with saline only demonstrated that CCL_2_ elevation in the sciatic nerve accompanied CX_3_CR_1_ deficiency (Fig. 4a, b). In addition, we found that two VCR cycles increased CCL_2_ expression in the sciatic nerve of both CX_3_CR_1_-deficient and heterozygous mice (Fig. 4a, b). Immunohistochemical analysis of GFP+ (macrophages) and CCL_2_+ profiles in the sciatic nerve suggested that increases in CCL_2_ in the sciatic nerve in CX_3_CR_1_-deficient mice and in both genotypes in response to VCR treatment were, at least in part, a result of an increase of CCL_2_ in macrophages (Fig. 4c, d). In order to confirm that the results obtained were not a misrepresentation afforded by potential antibody artifacts, we confirmed that CCL_2_ could not be detected in sciatic nerve tissue from CCL_2_-deficient mice (data not shown).

### CX_3_CR_1_ deficiency is associated with high CCR_2_^+^ monocyte infiltration in peripheral nerves

Our data to this point demonstrates that (i) treatment with a CCR_2_ antagonist (RS-102895) reduces VCR-induced allodynia and monocyte/macrophage infiltration in sciatic nerves in CX_3_CR_1_-deficient mice during cycle two specifically and (ii) VCR induces CCL_2_ elevation in monocytes/macrophages. However, whether VCR allodynia is associated with peripheral nerve infiltration of CCR_2_-expressing monocytes, which provide a cellular target for RS-102895, has yet to be determined. We therefore considered evaluating monocytes/macrophages in peripheral nerve tissue specifically; however, the yield obtained during isolation was suboptimal for reliable characterisation. We therefore used peritoneal lavages as a surrogate source of monocytes [9]. Importantly, we confirmed that CCR_2_ receptor is indeed detectable in both control and lipopolysaccharide (LPS)-stimulated peritoneal cells using both Western blot and FACS analysis of Ly6C^+^ CCR_2_^+^ cells (Additional file 1: Figure S4).

In light of our evidence that CCR_2_ receptor is detectable, we measured peritoneal CCR_2_^+^ monocytes (F4/80^−^ cells) in CX_3_CR_1_-deficient and heterozygous mice following two cycles of VCR using FACS analysis. We observed that in both genotypes, two cycles of VCR treatment significantly increased F4/80^−^Ly6C^+^CCR_2_^+^ cells relative to saline-treated controls (Fig. 4e, f). Furthermore, the number of F4/80^−^Ly6C^+^CCR2^+^ cells in VCR-treated CX_3_CR_1_-deficient mice was significantly greater than that measured in heterozygous controls, indicating that CX_3_CR_1_ deficiency was associated with an increase of CCR2^+^ monocyte numbers in vivo. Critically, treatment with RS-102895 during the second VCR cycle significantly reduced the number of F4/80^−^Ly6C^+^CCR_2_^+^ cells to a greater extent in CX_3_CR_1_-deficient mice (Fig. 4e, f). Taken together, this data is suggestive of an interaction between CX_3_CR_1_ and CCR_2_ receptors in monocytes.

### Knocking down CX_3_CR_1_ expression in a human monocyte cell line results in an increase in CCL_2_/R_2_ expression via p38 MAPK signalling

**Fig. 5:**
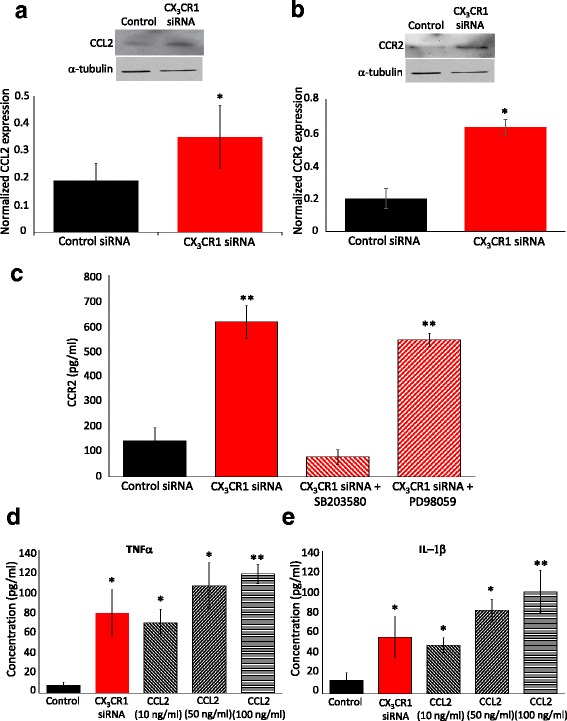
Downregulation of CX_3_CR_1_ increases the expression of CCL_2_/R_2_ and proinflammatory cytokines TNFα and IL-1β. **a** Representative Western blot and quantification for CCL_2_ (15 kDa) in control siRNA and CX_3_CR_1_ siRNA-transfected THP-1 cells (mean ± SEM, *n* = 3 cultures, three wells per culture were pooled). Expression of CCL_2_ is significantly elevated in CX_3_CR_1_ siRNA-transfected THP-1 cells relative to controls. *p < 0.01, Student’s *t* test. **b** Representative Western blot and quantification for CCR_2_ (42 kDa) in control and CX_3_CR_1_ siRNA-transfected THP-1 cells ± pre-treatment with either SB203580 (p38 MAP Kinase inhibitor) or PD98059 (MEK inhibitor). **c** Quantification of CCR_2_ expression (pg/ml) using ELISA (mean ± SEM, *n* = 3 cultures). Transfection of THP-1 cells with CX_3_CR_1_ siRNA with PD98059 pre-treatment results in a significant increase in CCR_2_ expression. This is not observed following pre-treatment with SB203580. **p < 0.01 relative to control siRNA transfected. **d**, **e** TNFα (**d**) and IL1β (**e**) quantification by ELISA of THP-1 culture medium following transfection with CX_3_CR_1_ siRNA or 3 h stimulation with recombinant CCL_2_ (10, 50 and 100 ng/ml). CX_3_CR_1_ downregulation and all concentrations of CCL_2_ significantly increase TNFα and IL1β expression (mean ± SEM, *n* = 3 cultures). *p < 0.05 and**p < 0.01 relative to control, Student’s *t* test

In order to investigate whether CX_3_CR_1_ regulates CCR_2_ expression in monocytes, we knocked down CX_3_CR_1_ expression using siRNA in the human monocyte THP-1 cell line, which resulted in an approximately 80% reduction in CX_3_CR_1_ expression (Additional file 1: Figure S5A). As shown previously [26], we confirmed that downregulation of the CX_3_CR_1_ receptor in monocytes is accompanied by an increase in CCL_2_ expression (Fig. 5a). In addition, however, a significant increase in CCR_2_ expression was also observed, with CX_3_CR_1_ siRNA-transfected THP-1 cells expressing significantly higher levels of CCR_2_ relative to those transfected with control siRNA (Fig. 5b, Additional file 1: Figure S4B). Furthermore, when cells were pre-treated with the p38 MAP kinase inhibitor, SB203580, downregulation of CX_3_CR_1_ (which is unaffected by SB203580 administration (Additional file 1: Figure S5C)) did not result in an increase in CCR_2_ expression (Fig. 5c). When cells were pre-treated with the MAPK/ERK kinase inhibitor, PD98059, however, CX_3_CR_1_ downregulation resulted in an increase in CCR_2_ expression (Fig. 5c). Taken together, these data suggest that downregulation of CX_3_CR_1_ in THP-1 cells in vitro results in upregulation of CCR_2_ receptor via p38 MAP Kinase signalling.

### Downregulation of CX_3_CR_1_ regulates proinflammatory cytokine levels in vitro

In order to begin to identify a mechanism by which an interaction between CX_3_CR_1_ and CCR_2_ in monocytes could potentially result in mechanical allodynia, we then went on to establish whether this interaction regulates the release of pronociceptive mediators. Indeed, downregulation of CX_3_CR_1_ resulted in a significant increase in the basal release of the proinflammatory cytokines TNF-α and IL1β to the same extent as stimulation of non-transfected THP-1 cells with 10 ng/ml CCL_2_ (Fig. 5d, e). Furthermore, release of TNF-α and IL1β, following stimulation with higher doses of CCL_2_ (50 and 100 ng/ml), was not significantly higher than those observed following CX_3_CR_1_ receptor downregulation alone (Fig. 5d, e).

In short, our THP-1 cell data demonstrates the presence of a novel interaction between CX_3_CR_1_ and CCR_2_ receptor expression, which is mediated by p38 MAP kinase signalling. In addition, it demonstrates that the CX_3_CR_1_-CCR_2_ interaction in vitro regulates the release of pronociceptive cytokines, thus providing a potential means by which monocytes could communicate with sensory neurons in the mediation of allodynia in CX_3_CR_1_-deficient mice during the second VCR cycle.

## Discussion

Chemotherapy-induced painful neuropathy (CIPN) is a dose-limiting side effect that jeopardises the success of cancer therapy [1]. The need for novel, more effective pain therapies, which necessitates a deeper understanding of the underlying mechanisms, is therefore profound. Different chemotherapeutic agents are likely to induce CIPN via a range of mechanisms, and thus, future therapies are likely to be most effective if they are tailored for specific cancer treatments.

In this study, we elucidate a mechanism underlying pain associated with vincristine (VCR) treatment and present evidence to suggest that CCR_2_ receptor signalling in monocytes infiltrating the sciatic nerve plays a role in VCR-induced allodynia in CX_3_CR1-deficient mice during the second cycle specifically. We begin to elucidate a novel interaction between CX_3_CR1 and CCR_2_ in monocytes and demonstrate in vitro that it regulates the release of proinflammatory cytokines that have been previously shown to modulate neuronal mechanisms underlying chronic pain [23, 29].

We initially show that in CCR_2_-deficient mice, VCR-induced allodynia and concurrent monocyte infiltration into the sciatic nerve develop to the same severity and within the same time frame as CCR_2_ heterozygous littermates but are both significantly reduced during the second VCR cycle. Our findings from CCR_2_-deficient mice however is not enough to confirm a role for CCR_2_ signalling in monocytes per se in VCR-induced pain. CCR_2_ mediates the egression of monocytes from the bone marrow into the bloodstream where they survey the environment surrounding tissue [30, 31]. It is therefore plausible that the reduction in allodynia that we observe is a result of prevention of monocyte egression as opposed to CCR_2_ signalling in macrophages in the sciatic nerve specifically. Indeed, pharmacological inhibition of CCR_2_ affords a very different scenario biologically to CCR_2_ deficiency and as such is accompanied by different behavioural and biological manifestations. Specifically, when we administered the CCR_2_ antagonist RS-102895 to VCR-treated mice during the second cycle, we observed that neither VCR-induced allodynia nor monocyte infiltration into the sciatic nerve was reduced.

Our pivotal observation however was that RS-102895 administration during the second VCR cycle did significantly reduce established VCR-induced allodynia and monocyte infiltration into the sciatic nerve of CX_3_CR_1_-deficient mice. Indeed, in keeping with this, CCR_2_ receptor inhibition in CX_3_CR_1_-deficient mice specifically has also been found to be protective in other pathological conditions such as macular degeneration [27].

In addition we found that the (i) VCR-induced increase in CCR_2_^+^ monocytes in the peritoneal cavity was exacerbated in CX_3_CR_1_-deficient mice and (ii) RS-102895 treatment significantly reduced the VCR-induced increase in CCR_2_+ monocytes in CX_3_CR_1_-deficient mice specifically. Taken together, these data are suggestive of an interaction between CX_3_CL_1_/CX_3_CR_1_ and CCL_2_/CCR_2_ chemokine/receptor systems in monocytes that could underlie the onset of VCR-induced allodynia in CX_3_CR_1_-deficient mice.

Interactions between CX_3_CR_1_ receptor and CCL_2_ have been reported previously in other contexts. Indeed, in addition to our observation that CCL_2_ is elevated in the sciatic nerve of CX_3_CR_1_-deficient mice, the expression of CCL_2_ has also been shown to be elevated in mononuclear phagocytes in the retina in CX_3_CR_1_-deficient mice [27]. Here, we present novel evidence that CCR_2_ receptor expression is also altered downstream of CX_3_CR_1_ downregulation in vitro. Specifically, when we downregulated CX_3_CR_1_ expression in immortalised human monocyte THP-1 cells, we observed a significant upregulation in both CCL_2_ and CCR_2_. Pharmacological inhibition of p38 MAPK but not MEK prevented this effect, and thus, we suggest that downregulation of CX_3_CR_1_ results in upregulation of the CCR_2_ receptor via p38 MAPK signalling.

As well as CX_3_CR_1_-mediated regulation or CCL_2_ and CCR_2_ expression in monocytes, the converse interaction has also been observed previously. Specifically, CCL_2_ stimulation of monocytes has been found to increase CX_3_CR_1_ receptor expression at monocyte membranes, most likely due to the stimulation of receptor translocation from intracellular stores as opposed to the stimulation of de novo expression [31]. This regulation has been shown to occur via CCR_2_-mediated activation of p38 MAPK, with pharmacological inhibition of p38 MAPK, attenuating the effect [31]. This data, in conjunction with the data presented in our study, suggests that the interaction between CX_3_CR_1_ and CCR_2_ receptors in monocytes is reciprocal and that expression of CX_3_CR_1_ and CCR_2_ is mutually regulated.

As well as CX_3_CR_1_ deficiency being associated with an increase in CCL_2_-CCR_2_ expression, VCR treatment was also found to increase CCL_2_. Elevated CCL_2_/CCR_2_ signalling in CX_3_CR_1_-deificient mice under basal conditions could explain the efficacy of RS-102895. The basal elevation of CCL_2_/CCR_2_ signalling alone in CX_3_CR_1_-deficient mice is not likely to be sufficient to cause allodynia as withdrawal thresholds did not vary between saline-treated CX_3_CR_1_-deficient and heterozygous mice. However, the potentially additive effect of CX_3_CR_1_ deficiency and two cycles of VCR on CCL_2_/CCR_2_ expression could indeed result in susceptibility to hypersensitivity. This could account for the onset of allodynia in CX_3_CR_1_-deficient mice at later stages of VCR treatment and the therapeutic benefit of CCR_2_ inhibition, which could also be attributed to increases in CCR_2_+ monocytes found in CX_3_CR_1_-deficient mice. Indeed, CCL_2_/R_2_ signalling in sensory neurons is a well-established mediator of pain. The elevation of CCR_2_^+^ monocytes and CCL_2_ expression in sciatic nerves that we observe in CX_3_CR_1_-deficient mice, in addition to VCR-induced increases in CCL_2_, is likely to cumulatively increase activity of the CCL_2_/CCR_2_ signalling axis in sensory neurons, thus activating pain pathways [32–34].

Whereas CX_3_CR_1_ is mainly found in monocytes/macrophages, the CCR_2_ receptor is expressed by other cell types. Elucidating the role of CCR_2_ signalling in monocytes/macrophages specifically is therefore a more intricate process than unravelling the role of CX_3_CR_1_ signalling in monocytes. For instance, the CCR_2_ receptor is also expressed in sensory neurons [17]. To attribute at least part of our observed effect of RS-102895 in CX_3_CR_1_-deficient mice to inhibition of CCR_2_ in monocytes specifically, we needed to consider a role for CCR_2_ inhibition in sensory neurons. A well-established downstream target of CCR_2_ signalling in neurons is p-ERK activation, which is associated with noxious activation of sensory neurons [25]. At the end of two cycles of VCR treatment, we found that p-ERK2 is elevated in DRG neurons in both CX_3_CR_1_-deficient and heterozygous mice. However, RS-102895 reduced VCR-associated p-ERK2 expression to the same extent in both genotypes despite the fact that allodynia is significantly reduced in CX_3_CR_1_-deficient mice. Although this does not rule out a role for CCR2-mediated neuronal activation in VCR allodynia, since thresholds observed in CX_3_CR_1_-deficient mice are still considered to be allodynic, it could suggest that other CCR_2_-mediated mechanisms are just as pivotal in regulating the *severity* of allodynia. Another key observation was that after the treatment was terminated, p-ERK2 activation in VCR-treated mice that also received RS-102895 was no longer reduced, regardless of the genotype. Crucially, however, allodynia was still significantly reduced in CX_3_CR_1_-deficient mice, despite measureable p-ERK2 activation. This could suggest one of two things: either the reduction in VCR allodynia in RS-102895-treated CX_3_CR_1_-deficient mice does not depend on the effect on neuronal activation or the dominant underlying mechanisms regulating VCR allodynia change as VCR pain evolves into ongoing after VCR treatment is terminated. Regardless of the explanation, however, our data suggests that there is scope for CCR_2_-mediated mechanisms that do not only require activation of CCR_2_ in sensory neurons to regulate VCR-associated allodynia in CX_3_CR_1_-deficient mice. CCR_2_ signalling in monocytes could be a candidate for such a mechanism.

**Fig. 6:**
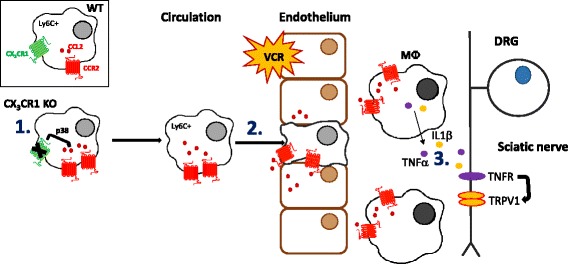
CCL_2_/CCR_2_ signalling in monocytes/macrophages plays a crucial role in VCR pain in CX_3_CR_1_-deficient mice. (1) Downregulation or silencing of CX_3_CR_1_ in monocytes results in upregulation of CCR_2_ via p38 MAP kinase signalling. (2) Monocytes infiltrate through the endothelium into peripheral nerve tissue in a CCL_2_/CCR_2_-dependent manner. (3) Cytokines such as TNFα and IL1β are released downstream of CCR_2_ signalling in monocytes. (4) TRPV_1_ channels mediate noxious signalling following TNFα receptor activation in sensory neurons

Indeed, our final, crucial observation was that downregulation of CX_3_CR_1_ in human monocytes in vitro regulated basal release of the proinflammatory cytokines TNFα and IL1β. It is well-established that both TNFα and IL1β are pronociceptive [29, 35], and thus, this provides us with a crucial link between the CX_3_CR_1_-CCR_2_ interaction in monocytes and noxious signalling. Indeed, we confirmed that CCL_2_ stimulation of THP-1 cells also results in TNFα and IL1β release and observed that both CCL_2_ and CCR_2_ are elevated in THP-1 cells when CX_3_CR_1_ is downregulated thus suggesting that downregulation of CX_3_CR_1_ results in the release of proinflammatory cytokines through heightened CCL_2_/CCR_2_ signalling. Our data suggests the presence of a novel interaction between CX_3_CR_1_ and CCR_2_ receptors, which regulates the release of monocyte-derived signals that mediate communication between monocytes and nociceptive neurons (Fig. 6). Critically, in vivo, we observe that under basal conditions, CX_3_CR_1_ deficiency is associated with an increase in CCL_2_ in macrophages in the sciatic nerve and that VCR treatment increases CCL_2_ expression further, which would cumulatively result in the release of pronociceptive cytokines and thus mediate pain signalling.

## Conclusions

Our data provides exciting evidence for a novel role of CCR_2_ receptor signalling in monocytes in VCR-associated allodynia in CX_3_CR_1_-deficient mice, which is likely to manifest as a result of an interaction between CX_3_CR_1_ and CCR_2_ expression in monocytes in the periphery. Identification of this interaction could thus uncover a new therapeutic target for longer-term treatment of vincristine pain.

## Additional file

Additional file 1: **Table S1.** Antibodies used for immunohistochemistry. **Table S2.** Antibodies used for Western blot analysis. **Figure S1.** No microglial response is detectable in VCR-treated CCR_2_ heterozygous or knockout mice. **Figure S2.** Prophylactic treatment of CX_3_CR_1_ heterozygous mice with RS-102895 does not affect the onset or severity of allodynia in cycle 1. **Figure S3.** Treatment with RS-102895 does not reduce VCR-associated allodynia in CX3CR_1_^+/GFP^ mice. **Figure S4.** Peritoneal monocytes/macrophages express CCR_2_ under basal conditions. **Figure S5.** Transfection of THP-1 cells with CX_3_CR_1_ siRNA downregulates CX_3_CR_1_ expression and upregulates CCR_2_ expression via p39 MAP kinase. (DOCX 960 kb)

## Abbreviations

CCL_2_: C-C motif chemokine ligand 2
CCR_2_: C-C chemokine receptor type 2
CIPN: Chemotherapy-induced painful neuropathy
CX_3_CL_1_: C-X3-C motif chemokine ligand 1
CX_3_CR_1_: C-X3-C motif chemokine receptor 1
IL1β: Interleukin 1-beta
MEK: MAPK/ERK kinase
p38 MAPK: P38 mitogen-activated protein kinase
p-ERK1/2: Phospho-p44/42 MAPK (Erk1/2)
siRNA: Short interfering ribonucleic acid
THP-1: Tamm-Horsfall protein 1
TNFα: Tumour necrosis factor alpha
TRPV_1_: Transient receptor potential cation channel subfamily V member 1
VCR: Vincristine
