# Supplementary material for: A novel interaction between CX3CR1 and CCR2 signalling in monocytes constitutes an underlying mechanism for persistent vincristine-induced pain
Source: J Neuroinflammation. 2018 Apr 6;15:101. doi: 10.1186/s12974-018-1116-6 (PMC5889528; doi:10.1186/s12974-018-1116-6)
Supplement: Supplementary file 1 — Table S1. Antibodies used for immunohistochemistry. Table S2. Antibodies used for Western blot analysis. Figure S1. No microglial response is detectable in VCR-treated CCR2 heterozygous or knockout mice. Figure S2. Prophylactic treatment of CX3CR1 heterozygous mice with RS-102895 does not affect the onset or severity of allodynia in cycle 1. Figure S3. Treatment with RS-102895 does not reduce VCR-associated allodynia in CX3CR1+/GFP mice. Figure S4. Peritoneal monocytes/macrophages express CCR2 under basal conditions. Figure S5. Transfection of THP-1 cells with CX3CR1 siRNA downregulates CX3CR1 expression and upregulates CCR2 expression via p39 MAP kinase. (DOCX 960 kb) [file 12974_2018_1116_MOESM1_ESM.docx]

**Supplementary Material**

**A novel interaction between CX_3_CR_1_ and CCR_2_ signalling in monocytes constitutes an underlying mechanism for long-term Vincristine pain.**

| **Antibody** | **Source** | **Dilution** |
| --- | --- | --- |
| **Primary** | | |
| Rabbit Iba1 (microglia) | WAKO | 1:1000 |
| Rat F4/80 (macrophages) | Abcam | 1:400 |
| Rabbit CCL2 | Invitrogen | 1:200 |
| Rabbit p-ERK | Cell Signalling Technology | 1:300 |
| **Secondary** | | |
| Anti-rabbit Alexa Fluor 488 | Invitrogen | 1:1000 |
| Anti-rabbit Alexa Fluor 568 | Invitrogen | 1:1000 |
| Anti-rat Alexa Fluor 488 | Invitrogen | 1:1000 |
| Anti-rat Alexa Fluor 568 | Invitrogen | 1:1000 |

**Table S1:** Antibodies used for immunohistochemistry

| **Antibody** | **Source** | **Dilution** |
| --- | --- | --- |
| **Primary** | | |
| Rat F4/80 (macrophages) | Abcam | 1:1000 |
| Rabbit MCP-1 (anti-mouse) | Abcam | 1:1000 |
| Rabbit MCP-1 (anti-human) | Abcam | 1:1000 |
| Rabbit CCR2 (anti-human) | Abcam | 1:1000 |
| Rabbit CX_3_CR1 (anti-human) | Abcam | 1:1000 |
| Rabbit p-ERK | Cell Signalling Technology | 1:1000 |
| Mouse α-tubulin | Abcam | 1:1000 |
| **Secondary** | | |
| HRP-rabbit | DAKO | 1:2000 |
| HRP-mouse | DAKO | 1:2000 |
| HRP-rat | DAKO | 1:2000 |

**Table S2:** Antibodies used for western blot analysis

**Supplementary figures and legends**

**
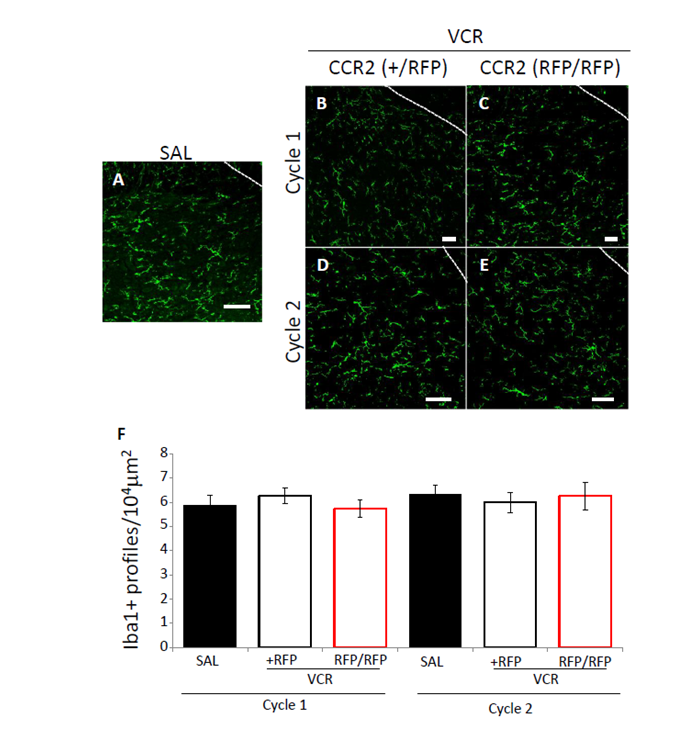
**

**Figure S1. No microglial response is detectable in VCR-treated CCR_2_ heterozygous or knock-out mice. (A)** Representative image of microglia (Iba1+, green) in lumbar spinal cord dorsal horn from saline-treated CCR_2_+/RFP mice. Scale bar = 30 μm. **(B-E)** Representative images of microglia in lumbar spinal cord dorsal horn transverse sections from CCR_2_+/RFP and CCR_2_^RFP/RFP^ mice during the first and second VCR cycle. Scale bar = 30 μm. **(F)** Quantification of the number of Iba1 immunoreactive (+) profiles in lumbar dorsal horn per 10^4^μm^2^ (mean ± SEM, n=4 mice per group). VCR does not result in a change in the number of Iba1 cells during either cycle in either genotype.

**Figure S2. Prophylactic treatment of CX_3_CR_1_-heterozygous mice with RS-102895 does not affect the onset or severity of allodynia in cycle 1.** The vincristine (VCR)-induced reduction in mechanical withdrawal threshold is not significantly dampened by prophylactic administration of RS-102895 during the first VCR cycle (days 0-4 inclusive).


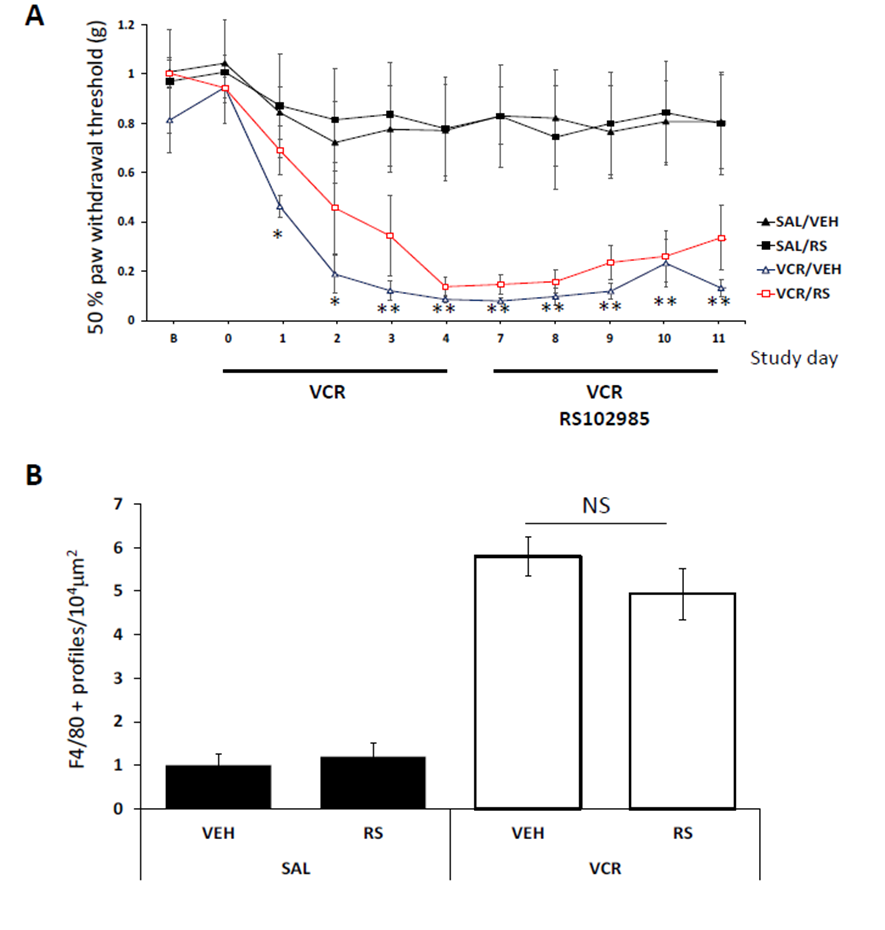


**Figure S3. Treatment with RS-102895 does not reduce VCR-associated allodynia in CX3CR_1_^+/GFP^ mice. (A)** CX_3_CR_1_-heterozygous (+/GFP) mice were treated with two VCR cycles (0.5 mg/kg/day i.p), alongside the CCR_2_ antagonist RS-102985 (20 mg/kg/day i.p), or vehicle, during the second VCR cycle. CX_3_CR_1_-heterozygous mice developed significant VCR-induced allodynia within 24 hours of VCR treatment (blue trace) as previously reported (mean ± SEM, n=6-9 mice per group). * and ** p < 0.05 and 0.01, respectively relative to saline-treated CX_3_CR_1_+/GFP mice, 2-way RM ANOVA, post-hoc Tukey test. When VCR-treated CX_3_CR_1_-heterozygous mice received RS-102985 (red trace), withdrawal thresholds did not change relative to VCR-treated CX_3_CR_1_-heterozygous mice who received vehicle (blue trace). **(B)** Quantification of immunohistochemical analysis of F4/80+ profiles (macrophages) per 10^4^ μm^2^. (Mean ± SEM, n=4 mice per group).

**
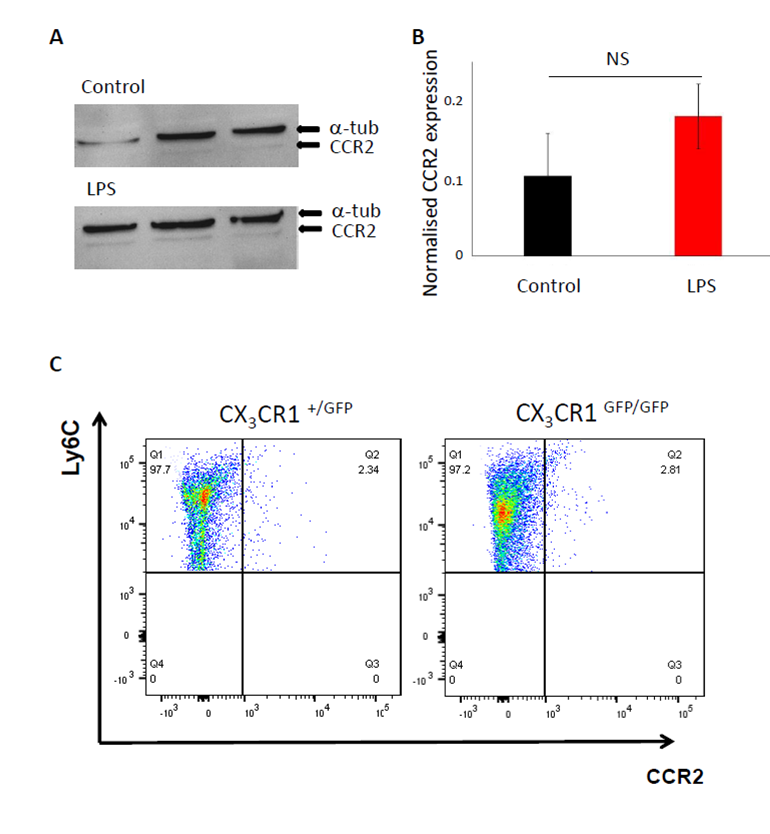
**

**Figure S4. Peritoneal monocytes/macrophages express CCR_2_ under basal conditions. (A)** Representative blot for CCR_2_ (43 kDa) and loading control α-tubulin (50 kDa) in non-stimulated (control) and LPS-stimulated peritoneal monocytes/macrophages. **(B)** Quantification of CCR_2_ band density normalised to α-tubulin (mean ± SEM, n=3). CCR_2_ is detectable in both control and LPS-stimulated peritoneal monocytes/macrophages, with no difference in expression between the two conditions. NS = not significant. **(C)** Representative dot blot from CX_3_CR_1_ heterozygous (+/GFP) and deficient (GFP/GFP). Ly6C^+^CCR_2_^+^ events are observed for both genotypes.


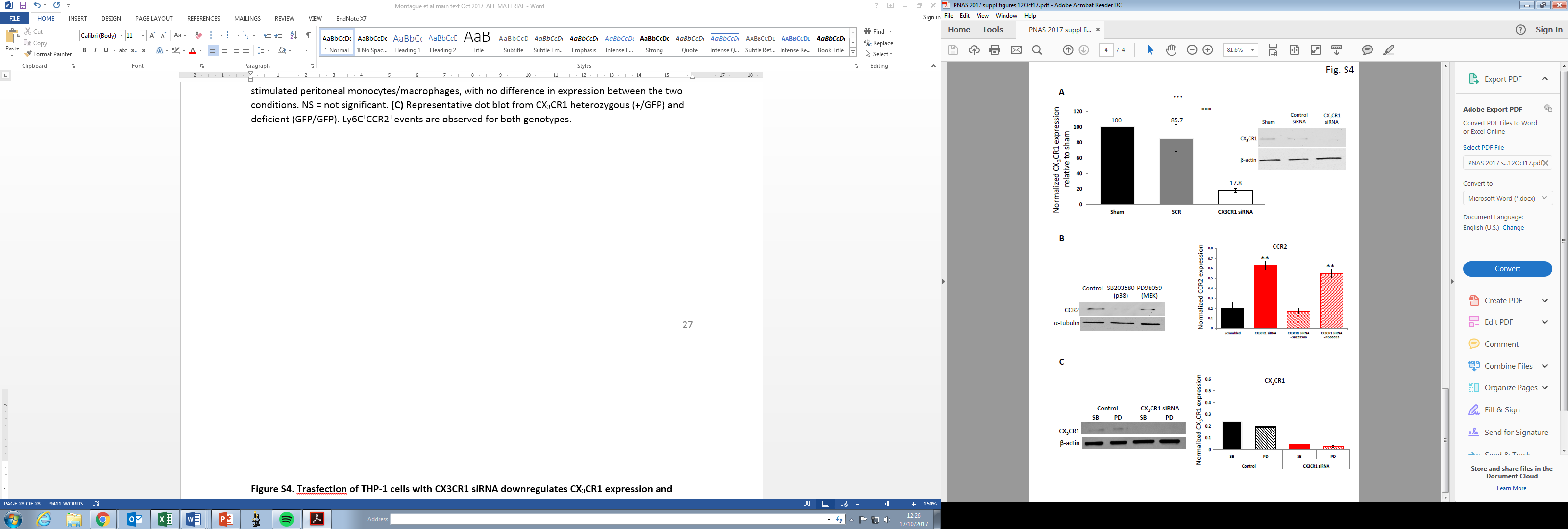


**Figure S5. Transfection of THP-1 cells with CX_3_CR_1_ siRNA downregulates CX_3_CR_1_ expression and upregulates CCR_2_ expression via p39 MAP Kinase. (A)** Representative blot for and quantification of CX_3_CR_1_ (50 kDa) in sham-transfected, control siRNA-transfected and CX_3_CR_1_ siRNA-transfected THP-1 cells (normalised to β-actin). CX_3_CR_1_ siRNA transfection reduces CX_3_CR_1_ expression by 82.2% (mean ± SEM, n=3). **(B)** Representative blot for and quantification of CX_3_CR_1_ (50 kDa) in control siRNA-transfected and CX_3_CR_1_ siRNA-transfected THP-1 cells (normalised to β-actin) in the absence or presence of SB203580 (p38 MAP Kinase inhibitor) or PD98059 (MEK inhibitor). Neither drug prevents CX_3_CR_1_ siRNA from downregulating CX_3_CR_1_. **(C)** Representative blot for and quantification of CCR_2_ (43 kDa) in CX_3_CR_1_ siRNA-transfected THP-1 cells (normalised to β-actin) under control conditions and with pre-treatment with SB203580 or PD98059 1 hour prior to siRNA transfection. Pre-treatment with SB203580 but not PD98059 prevents upregulation of CCR_2_ downstream of CX_3_CR_1_ downregulation (mean ± SEM, n=3). ** p < 0.01, Student’s t-test.
